# Theoretical discrimination index of postural instability in amyotrophic lateral sclerosis

**DOI:** 10.1038/s41598-022-06471-6

**Published:** 2022-02-14

**Authors:** Rodolphe Vallée, Alexandre Vallée, Jean-Noël Vallée, Malek Abidi, Annabelle Couillandre, Nicolas Termoz, Pierre-François Pradat, Giovanni de Marco

**Affiliations:** 1 Interdisciplinary Laboratory in Neurosciences, Physiology and Psychology: Physical Activity, Health and Learning (LINP2), UPL, Paris Nanterre University, Nanterre, France; 2 Laboratory of Mathematics and Applications (LMA) CNRS, UMR7348, DACTIM-MIS, Poitiers University, Poitiers, France; 3 Clinical Research and Innovation Department, Foch Hospital, Suresnes, France; 4 Neuroradiology Department, Amiens University Hospital, Picardie Jules Verne University, 80 000 Amiens, France; 5 COMUE Paris Lumières University, Paris, France; 6 Neurology Department, Reference Center for Amyotrophic Lateral Sclerosis, APHP, Pitié-Salpêtrière University Hospital, Paris, France; 7 Biomedical Imaging Laboratory, CNRS, INSERM, Sorbonne University, Paris, France; 8 Computational Neuroimaging Group, Trinity College Dublin, Dublin, Ireland; 9 Northern Ireland Centre for Stratified Medicine, Biomedical Sciences Research Institute Ulster University, C-TRIC, Altnagelvin Hospital, Londonderry, UK

**Keywords:** Medical research, Neurology

## Abstract

To assess the usefulness of a theoretical postural instability discrimination index (PI_th_) in amyotrophic lateral sclerosis (ALS). Prospective regression analyzes were performed to identify the biomechanical determinants of postural instability unrelated to lower limb motor deficits from gait initiation factors. PI_th_ was constructed using a logit function of biomechanical determinants. Discriminatory performance and performance differences were tested. Backward displacement of the pression center (APA_amplitude_) and active vertical braking of the mass center (Braking-index) were the biomechanical determinants of postural instability. PI_th_ = − 0.13 × APA_amplitude_ − 0.12 × Braking-index + 5.67, (*P* < 0.0001, RSquare = 0.6119). OR (APA_amplitude_) and OR (Braking-index) were 0.878 and 0.887, respectively, i.e., for a decrease of 10 mm in APA_amplitude_ or 10% in Braking-index, the postural instability risk was 11.391 or 11.274 times higher, respectively. PI_th_ had the highest discriminatory performance (AUC 0.953) with a decision threshold value $\ge$ 0.587, a sensitivity of 90.91%, and a specificity of 83.87%, significantly increasing the sensitivity by 11.11%. PI_th_, as objective clinical integrator of gait initiation biomechanical processes significantly involved in dynamic postural control, was a reliable and performing discrimination index of postural instability with a significant increased sensitivity, and may be useful for a personalized approach to postural instability in ALS.

## Introduction

Amyotrophic lateral sclerosis (ALS) is a progressive multisystem neurodegenerative disease that inexorably progresses and primarily affects the pyramidal motor system involving the motor cortex, cranial nerve motor nuclei and spinal cord motor neurons with additional evidence of non-motor functional involvements^1–4^. ALS may cause cognitive-behavioral, extrapyramidal and sensory disturbances by progressive extension of degeneration to the corresponding brain structures. There is accumulating clinical^5^, neuroimaging^6–8^ and postmortem neuro-pathological^9^ evidence of extrapyramidal involvement in patients with ALS^9,10^.

In addition to motor weakness and spasticity, extrapyramidal involvement in ALS may contribute to postural system impairments with risks of falls and fractures affecting quality-of-life and mortality^5,10^. Postural instability can be an early functional sign preceding motor weakness, especially in patients with upper motor neuron predominance, as reported by our team^5,11^ and others^10,12,13^. Extrapyramidal stiffness is correlated with postural and balance disorders in ALS patients^5^. However, the conjunction of motor neuron features as well as potential extrapyramidal, cerebellar^14^ and vestibular^15^ disorders in ALS does not allow an accurate clinical evaluation of postural instability and gait impairment, which requires quantitative assessment approaches.

Postural stability is the dynamic postural response to applied or volitional perturbations^16^, and is an essential component in assessing the effectiveness of interventions to improve balance^17,18^. Gait initiation is an interesting pattern to investigate dynamic postural instability related to different neurological disorders such as ALS. It is the motor transition from standing position to walking. It includes an anticipatory postural adjustment (APA) phase preceding the swing foot-off*,* followed by the step execution phase^19,20^. APAs result in a backward shift in the center of pressure (CoP)^19^ toward the swing leg side, which allows the propelling of the center of mass (CoM) toward the stance leg side prior to the swing heel-off^21,22^. APAs create the conditions which act to propel the CoM forward contributing to body forward progression to reach the intended gait speed at the first step end^19,23^, and the optimal conditions needed to maintain the stability of the whole body during step execution^24–26^.

Postural instability is usually assessed clinically using the pull test, which is a common approach in Parkinson’s disease^27^. However, the inter-examiner reliability of pull test may be considered suboptimal relying on some degree of subjective interpretation by examiners, which may provide an inadequate assessment of postural instability^27^.

Clustering is a multivariate technique of grouping individuals sharing similar values across several variables in which the data is usually not scattered evenly through n-dimensional space but instead forms clusters. Identifying these clusters may make sense of this clumped data and may provide a deeper understanding of data. Thus, cluster analysis of subjects across gait initiation and postural instability variables may be regarded as an alternative or complementary approach to the pull test, to the extent to which methods collecting data may assign the same score to the same postural instability variable.

The aim of the study was to identify the biomechanical determinants of postural instability unrelated to lower limb motor deficits from gait initiation factors in ALS. Then with a view to increase discriminatory performance, according to a personalized approach to postural instability, the purpose was to create a theoretical postural instability discrimination index (PI_th_) using multivariable regression models from the identified biomechanical determinants, evaluate its usefulness and added discriminatory performance value. Finally, to assess the inter-method reliability between the pull test and clustering of subjects, across the gait initiation and postural instability variables, in order to highlight clusters of data in relation to postural instability.

## Methods

The design of this experimental prospective study was previously described^11^. 45 subjects were recruited in the study, 31 patients fulfilling the El Escorial criteria for probable or definite ALS with no motor deficit in the lower limbs, and 14 age- and gender-matched healthy controls. Of the 31 ALS patients, 14 had postural instability and 17 had none, based on the clinical pull test^28^. The demographic characteristics of the ALS patients are summarized in Table 1. In 5 patients, postural instability and falls were the initial symptoms, 3 of them showing a neurogenic pattern in the lumbar region at EMG but with no detectable lower limb weakness. The study subjects (ALS patients and controls) were classified into 2 groups with (14), and without (31) postural instability, based on the pull test.

**Table 1:** The demographic and clinical profile of study participants.

	Healthy controls (*n* = 14)	ALS with postural instability (*n* = 14)	ALS without postural instability (*n* = 17)	*p* value
Age (years)	63.0 (57.0–66.0)	59 (57–62)	58.0 (50.0–64.0)	0.55
Gender (female/male)	5/9	3/11	6/11	0.40
Height (cm)	170 (168–175)	170 (161–176)	171(165–178)	0.51
Weight (kg)	74.5 (66.0–83.7)	67.2 (64.2–85.7)	72.0 (56.0–80.0)	0.79
**Disease onset (type)**				
Postural instability	N/A	3	2	0.47
Upper limb weakness		6	10	0.38
Bulbar symptoms		5	5	0.71
ALSFRS-r (max 48)	N/A	37.5(35.2–41.0)	4 1.0 (38.0–43.0)	0.09
ALSFRS-R1 bulbar (max 12)		1 1.0 (10.2–12.0)	1 2.0 (07.0–12.0)	0.55
ALSFRS-R2 upper Limb (max 12)		10.0 (07.0–11.0)	08.0 (05.0–11.0)	0.53
ALSFRS-R3 low Limb (max 12)		07.0 (05.2–07.7)	11.0 (09.0–12.0)	0.70
ALSFRS-R4 respiration (max 12)		12.0 (12.0–12.0)	12.0 (12.0–12.0)	0.64
Disease duration (months)	N/A	23.5(14.7–37.2)	17.0 (12.0–27.0)	0.20
Disease progression rate (months)	N/A	0.44 (0.19–0.78)	0.42 (0.32–0.05)	0.92
Cognitive assessment				
**California verbal learning test II CVLT II**				
Immediate recall	N/A	07.0 (05.0–08.0)	07.0 (06.0–09.0)	0.79
total trial recall (1–5)		53.5 (51.2–58.7)	57.0 (51.0–63.0)	0.87
Short delay free recall		13.0 (11.2–13.7)	12.0 (11.0–14.0)	0.48
Short delay cued recall		01.5 (01.0–12.0)	02.0 (01.0–04.0)	0.37
Long delay free recall		14.0 (13.0–14.7)	14.0 (13.0–15.0)	0.88
Long delay cued recall		01.5 (01.0–12.7)	01.0 (00.0–02.0)	0.68
Total recognition discrimination		16.0 (16.0–16.0)	16.0 (15.0–16.0)	0.41
**Stroop test**				
Reading	N/A	99.0 (87.0–103.0)	98.0 (87.0–109.0)	0.82
Naming		72.0 (63.0–077.0)	70.0 (59.2–74.75)	0.90
Double task		38.0 (31.0–042.0)	37.5 (34.0–43.7)	0.56
**Verbal fluency test**				
Phonemic	N/A	22.0 (17.2–27.0)	20.0 (17.0–31.0)	0.85
Semantic		31.0 (26.0–32.0)	35.0 (22.0–42.0)	0.60
**Wisconsin card sorting test**				
Categories achieved	N/A	06.0 (04.0–06.0)	06.0 (05.5–06.0)	0.35
Perseverative errors		09.5 (07.0–11.7)	07.0 (05.0–10.5)	0.10
		03.5 (01.2–06.7)	03.0 (01.0–04.0)	0.21
**Digit span**				
Forward	N/A	08.0 (07.0–09.0)	09.0 (07.0–12.0)	0.08
Backwards		04.5 (04.0–07.0)	06.0 (04.0–08.0)	0.38

Values presented as median (range) for functional scores followed by the minimum and the maximum values. ALSFRS-r: The revised ALS functional rating scale. N/A: not applicable. Disease progression = (48 − ALSFRS-R scale/disease duration) (Ref. Ferron et al.^11^).

The study was approved by the local ethics committee approval Ile-de-France CPI Paris VI, *Institut National pour la Santé et la Recherche Médicale* (INSERM): approval number RBM C12-13. All participants gave their informed written consent in conformity with the Declaration of Helsinki.

### Clinical pull test

The pull test execution was standardized and performed by the same experienced neurologist (PFP). The patient was standing with eyes open and feet comfortably apart. The examiner stood behind the patient and applied a brisk and forceful pull on the patient's shoulders, while respecting the safety conditions for the patient, while the examiner stood ready to catch the patient to prevent its fall. The patient had to prevent himself from falling, if necessary, by taking a step backward after being pulled.

The postural instability scores ranged from 0 to 4 (0: normal recovery; 1: retropulsion, unaided recovery; 2: no postural response (no recovery), fall if not caught by the examiner; 3: very unstable, tendency to lose balance spontaneously; 4: unable to stand without assistance).

Score of 1 or more was classified into the postural instability group, and 0, in the group without postural instability.

### Kinematic and kinetic recordings of gait initiation

The gait initiation test was performed in natural gait conditions using a force plate (0.9 × 1.8 m, AMTI, Advanced Mechanical Technology Inc. Watertown, MA, USA). Twenty consecutive trials were recorded for each subject^11^. From the biomechanical data records of gait initiation, accelerations, velocities, displacements of the CoM, and displacements of the foot CoP were computed during the first two steps^19^. By convention, accelerations, velocities, and displacements were considered positive when directed forward, upward, and toward the swing leg side.

### Data analysis

The gait initiation biomechanical variables in the APA and execution phases were analyzed. APA_duration_ (s) was the time between the backward movement start of the CoP and the swing foot-off*.* EP_duration_ (s) was the time between the swing foot-off and the stance foot-off. APA_amplitude_ (mm) was the maximal anteroposterior backward displacement of the CoP. L (mm) was the anteroposterior step length between the CoP position at the swing foot-off time and the stance foot-off time. V_m_ (m s^−1^) was the maximal anteroposterior progression velocity of the CoM at the end of the first step^19^, V_1_ (m s^−1^) and V_2_ (m s^−1^), the vertical downward V-shaped speed of the CoM with the minimal negative falling speed and the reduced falling speed at foot-contact time with the ground, respectively, during step execution. The Braking-index $\times 100$ (%) characterizes the active braking of the CoM vertical fall and reflects the dynamic postural control during step execution. A Braking-index below 25% was considered abnormal.

### Statistical analysis

The variables tested were the continuous biomechanical variables of gait initiation and the postural instability categorical variable dichotomized into with and without postural instability categories. Mean values and frequencies were expressed with their standard deviations (± SDs) and percentages (%), respectively.

Differences in values of biomechanical parameters between the subject groups with and without postural instability were assessed using the non-parametric U Mann–Whitney test for quantitative variables.

Univariate and multivariate multiple regression analyses were performed to evaluate the biomechanical parameters effects of APA phase on motor performances of step execution.

Multivariate logistic regression analyses with mixed stepwise selection were performed to identify potential gait initiation factors that were independently associated with postural instability. The maximum threshold of the p value was 0.25 for an effect to be able to enter the model during a forward step, and the minimum threshold was 0.10 for an effect to be removed from the model during a backward step.

Receiver operating characteristic (ROC) analysis was performed for individual gait initiation classifiers regarding their discriminatory ability for postural instability. For each classifier, the ability of the logistic regression models to allow discrimination was quantified by the area under the ROC curve (AUC). The maximum Youden index, *J* = max_c_ [Se(c) + Sp(c) − 1], was chosen to determine the optimal decision thresholds (c) for the discrimination.

PI_th_ was constructed using a multivariable logit function of the biomechanical classifiers of postural instability and modeled as follows:

$${\text{PI}}_{{{\text{th}}}} = {\text{b}}_{0} + {\text{b}}_{{1}} {\text{X}}_{{1}} + {\text{ b}}_{{2}} {\text{X}}_{{2}} + \cdots + {\text{b}}_{{\text{i}}} {\text{X}}_{{\text{i}}} ,$$

X_i_ were the gait initiation classifiers as effects of the model, i, the number of classifiers, b_i,_ the parameters estimated using the maximum likelihood estimation, and PI_th,_ the maximum likelihood estimate ranging from (−) infinite to (+) infinite.

Odds ratios with confidence intervals were calculated for the postural instability responses.

For given values of gait initiation parameters, the odds of postural instability were:

$${\text{Odds}}\left( {\text{postural instability}} \right) = {\text{ e}}^{{({\text{b}}0 \, + {\text{ b1X1 }} + {\text{ b2X2 }} + \cdots + {\text{ biXi}})}} = {\text{ e}}^{{{\text{b}}0}} {\text{e}}^{{{\text{b1X1}}}} {\text{e}}^{{{\text{b2X2}}}} \ldots {\text{ e}}^{{{\text{biXi}}}} ,$$

The unit odds ratio for the classifier X_i_, the co-variables being determined, was therefore,

$${\text{OR}}\left( {{\text{X}}_{{\text{i}}} } \right) = {\text{e}}^{{{\text{b}}0}} {\text{e}}^{{{\text{b1X1}}}} {\text{e}}^{{{\text{b2X2}}}} \ldots {\text{ e}}^{{{\text{bi}}({\text{Xi }} + {1})}} )/{\text{e}}^{{{\text{b}}0}} {\text{e}}^{{{\text{b1X1}}}} {\text{e}}^{{{\text{b2X2}}}} \ldots {\text{e}}^{{{\text{biXi}}}} = {\text{ e}}^{{{\text{b}}0}} {\text{e}}^{{{\text{b1X1}}}} {\text{e}}^{{{\text{b2X2}}}} \ldots {\text{e}}^{{{\text{biXi}}}} {\text{e}}^{{{\text{bi}}}} /{\text{e}}^{{{\text{b}}0}} {\text{e}}^{{{\text{b1X1}}}} {\text{e}}^{{{\text{b2X2}}}} \ldots {\text{e}}^{{{\text{biXi}}}} = {\text{e}}^{{{\text{bi}}}} .$$

Lack-of-fit tests were performed to address whether there was enough information using the selected variables in the fitted model for postural instability discrimination. The significance of the model effects, as a whole, for the statistical adjustment of postural instability discrimination was assessed using the likelihood-ratio Chi-square tests (whole model tests). The usefulness of models was measured by RSquare for logistic regression. The significance of the contribution of each effect to the adjustment of the model for postural instability discrimination was assessed using the effect likelihood ratio tests.

Next, ROC analysis was performed for the PI_th_ classifier using the logistic regression multivariate effect likelihood ratio test^29^ to assess the discriminatory performance (AUC) for postural instability. Differences in discriminatory performance of classifiers were tested, for significance purposes, by comparing the AUC values ​using bivariable Chi2 tests.

K-Means clustering was used to cluster the subjects together that share similar values across the gait initiation and postural instability variables. The number of clusters, K, was specified a priori. The optimal number of clusters providing the best fit was selected using the highest cubic classification criterion (CCC). The inter-method reliability between pull test and K-means clustering was assessed using the Kappa statistic.

SAS 9.1 (SAS Institute Inc., Cary, NC) was used for statistical analysis (2-sided and *P* value < 0.05).

## Results

### Gait initiation biomechanical parameters

Mean values (± SDs) of biomechanical parameters are shown in Table 2.

**Table 2:** Mean values (± SDs) of biomechanical parameters between the groups with and without postural instability.

Biomechanical parameters	Postural instability				Relative differences (%)*	*P* value**
	Without		With			
	Mean	Std Dev	Mean	Std Dev		
APA_duration_ (s)	0.566848832	0.061281525	0.641542953	0.086230476	− 13.18	0.0163
EP_duration_ (s)	0.537647635	0.055751953	0.537955033	0.086750354	− 0.05717	0.9772
(APA + EP)_duration_ (s)	1.1044964672	0.0841442296	1.1794979861	0.1300469655	− 06.79	0.1156
APA_amplitude_ (mm)	47.03460265	13.719062151	25.36872678	12.51979091	46.06	0.0003
V_m_ (m/s)	1.07374776	0.328735433	0.635699222	0.202091494	40.79	0.0004
L (m)	608.3422665	108.137478	448.4566466	96.84432282	26.28	0.0004
V_1_ (m/s)	− 0.15120231	0.0427494097	− 0.100879025	0.0321864512	33.28	0.0015
V_2_ (m/s)	− 0.086672738	0.043742405	− 0.09327473	0.0283038221	− 07.617	0.6678
Braking index (%)	41.384235781	26.021641604	6.7557709681	6.8640314088	83.67	0.0001
PI_th_	− 5.416201044	3.8196657635	1.5574518342	1.9792025093	128,76	0.0001

*In percentage of biomechanical parameter values without postural instability: ((without – with) / without) × 100.

**U Mann–Whitney test.

Subjects with postural instability, the values of biomechanical parameters APA_amplitude_, V_m_, L, V_1_ and Braking-index were significantly lower (*P* = 0.0003, 0.0004, 0.0004, 0.0015, and 0.0001, respectively), and APA_duration_, significantly higher (*P* = 0.0163), compared to subjects with no postural instability (Table 2). The decrease in APA_amplitude_ was significantly associated with a decrease in step length (L) and CoM forward progression velocity (V_m_) (*P* = 0.0041 and < 0.0001, respectively).

### Accuracy of individual biomechanical classifiers

**Figure 1:**
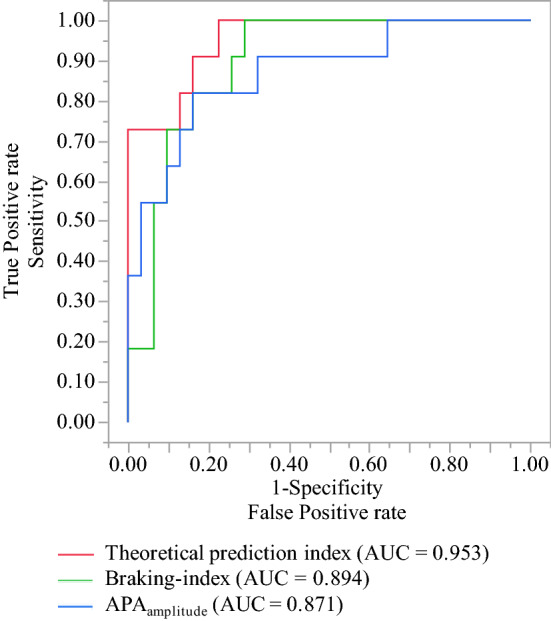
Discriminatory performances (AUCs obtained from ROC curves) of the biomechanical classifiers of gait initiation and the theoretical discrimination index of postural instability (PI_th_) classifier, and the differences in discriminatory performance of classifiers.

The AUCs obtained from ROCs and optimal decision thresholds for APA_amplitude_ and Braking-index classifiers are shown in Table 3; Fig. 1. The determinants of postural instability were APA_amplitude_ and Braking-index parameters (*P* < 0.0001, respectively). Their values significantly decreased by 46.06% and 83.67% (*P* = 0.0003 and 0.0001), respectively, in subjects with postural instability compared to those with no postural instability.

**Table 3:** Biomechanical classifiers of postural instability.

Biomechanical predictors	AUC	*P* value	Thresholds	Sensitivity	1-Specificity
APA_amplitude_ (mm)	0.87097	< 0.0001	$\le$ 31.6734	0.8182	(83.87%) 0.1613
Braking index (%)	0.89443	< 0.0001	$\le$ 8.04636	0.8182	(83.87%) 0.1613
PI_th_ (%) *	0.95308	< 0.0001	$\ge$ 0.58689	0.9091	(83.87%) 0.1613

*Theoretical prediction index classifier of postural instability.

Their accuracies were not significantly different (AUC = 0.871, 0.894, respectively, P = 0.82) for postural instability discrimination. Their optimal decision threshold values for postural instability discrimination were $\le$ 31.6734 and $\le$ 8.04636, respectively, with a sensitivity of 0.8182, and a specificity of 83.87%, respectively.

### Multi-fitted theoretical postural instability discrimination index

PI_th_ was modeled by the equation:

$${\text{PI}}_{{{\text{th}}}} = - 0.{13} \times {\text{APA}}_{{{\text{amplitude}}}} - 0.{12} \times {\text{Braking - index}} + {5}.{67};$$

with *P* < 0.0001 and RSquare = 0.6119 for the whole-model effects, *P* = 0.9975 for the lack of fit Chi-square of the whole-model, and *P* = 0.0200, 0.0235 and 0.0156 for the parameter estimates and intercept, respectively.

PI_th_ value significantly increased by 128.76% (*P* < 0.0001) in subjects with postural instability compared to those with no postural instability (Table 2). The contribution of each effect APA_amplitude_ and Braking-index in the adjustment of the model for postural instability discrimination was significant (*P* = 0.0016 and 0.0005, respectively). The unit odds ratios OR (APA_amplitude_) and OR (Braking-index) for postural instability were 0.8779 (95% CI 0.7593; 0.9591) and 0.8870 (95% CI 0.7745; 0.9611), respectively.

### Accuracy of the theoretical discrimination index classifier and discriminatory performance differences of classifiers for postural instability

The optimal decision threshold value of PI_th_ for postural instability discrimination was $\ge$ 0.58689 (*P* < 0.0001), with a sensitivity of 90.91%, and a specificity of 83.87%, calculated with the optimal decision threshold values of parameters APA_amplitude_ and Braking-index (Table 3).

PI_th_ had the highest discriminatory performance with significantly higher accuracy than the classifiers APA_amplitude_ and braking-index (AUC = 0.953 versus 0.871 and 0.894; *P* < 0.001 and 0.02, respectively), thus significantly increasing the sensitivity of discriminatory performance by 11.11%, i.e., from 81.82 to 90.91% (Table 3; Fig. 1).

### Pull test and K-means cluster analysis

**Figure 2:**
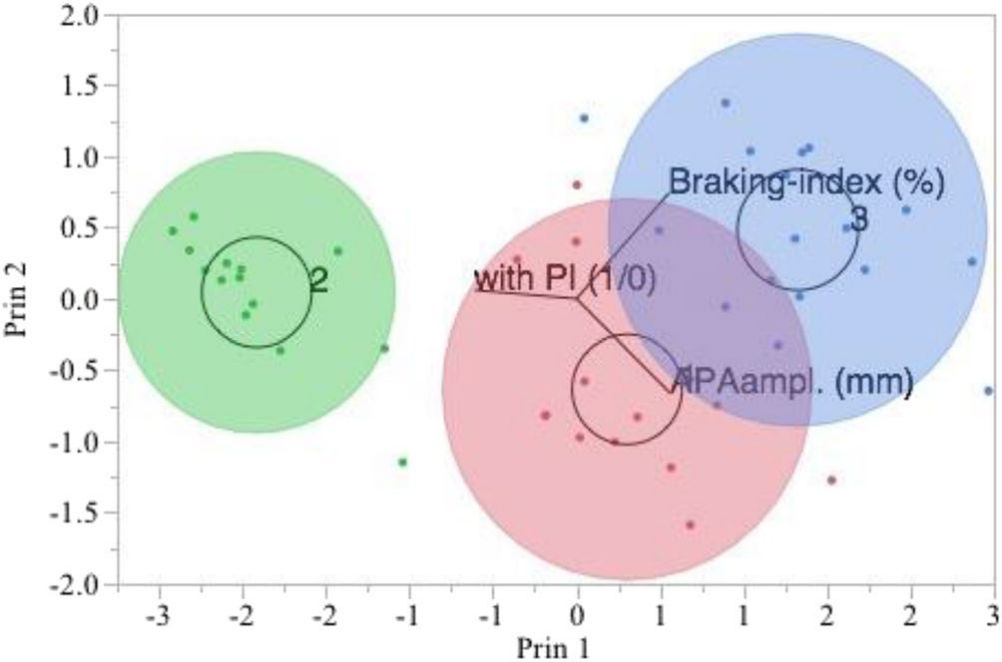
K-Means clustering method. Biplot for parameters of gait initiation and postural instability. Clusters 2, based on the parameters of gait initiation (APA_amplitue_ and Braking-index) and postural instability, stands out from the others. This is supported by its parallel coordinate plot in Fig. 3(2), which differs from the plots for the other clusters.

**Figure 3:**
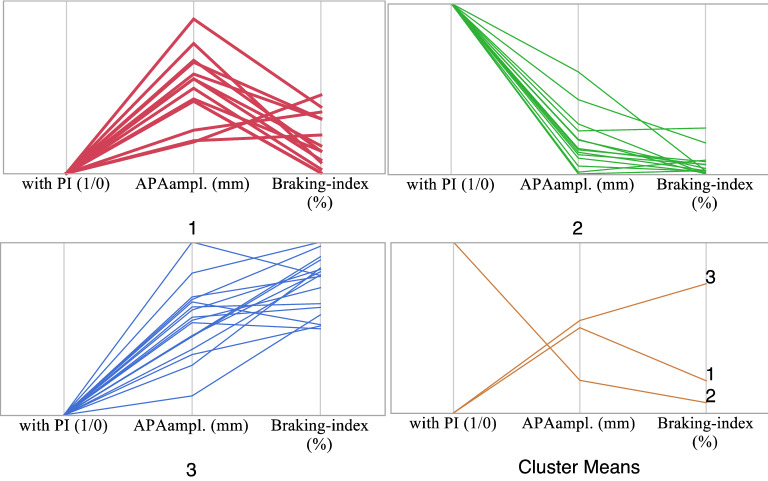
K-Means clustering method. Parallel coordinate plots for the display of the structure of the observations in each cluster showing how the clusters differ. Cluster 2 tends to have comparatively low APA_amplitude_ and Braking-index values and postural instability

K-means clustering across the gait initiation and postural instability variables was implemented with 3 clusters providing the best fit with the optimal CCC value of 4.85. It highlighted a subject cluster with postural instability and low values of APA_amplitude_ and Braking-index parameters appearing to be be standing out from others (Table 4; Figs. 2, 3). Agreement between K-means clustering and pull test was complete with Kappa coefficient of 1.

**Table 4:** K-Means clustering method.

Cluster means			
Cluster	Postural instability	APA_amplitude_ (mm)	Braking index (%)
1	0	45.445855	16.5675477
2	1	24.736571	6.35800396
3	0	48.342983	61.8215083

K cluster = 3, CCC best = 4.82779, Step = 5

Eigenvalues 2.996682, 0.5347658, 0.4685522, 0

## Discussion

Identifying and measuring the biomechanical determinants of postural instability from gait initiation factors can provide clinicians with objective measures to assess the effectiveness of rehabilitation programs and better target interventions, according to individual impairments. Determining the equations of logit functions of gait initiation determinants, using multivariate logistic regression models was aimed in order to model the probabilities of having postural instability. The stepwise variable selection procedure was used to interactively select a subset of variables which provide a good fit for the model, given there is little theory in literature to guide the selection of terms of a model for postural instability discrimination. The chosen direction of variable selection that enter and leave the model was a mixed direction that alternates the forward and backward steps. It includes the most significant term that satisfies the maximum p-value threshold and removes the least significant term satisfying the minimum P value threshold. It continues removing terms until the remaining terms are significant and then it changes to the forward direction.

To improve model discriminatory performance, the classifier combination strategy assumed conditional independence of the classifiers for the purpose of designing a theoretical discrimination index. It was based on the calculation of a combined ROC curve using the logistic regression multivariate effect likelihood ratio tests to determine a combination rule for each ROC operating point, which improved model discriminatory performance the most. The strategy was to reduce the variance caused by estimating unnecessary variables, thus reducing the number of variables in the model without significantly losing information from the whole variables studied; in other words, without significantly reducing the variance in postural instability explained by the whole variables studied.

Therefore, multi-fitted theoretical discrimination index of postural instability (PI_th_) was designed as a theoretical integrator of the biomechanical determinants of postural instability, from gait initiation factors in the APA and execution phases, to increase discriminatory performance of postural instability. It was conceived on the basis of a nomogram of the postural instability established from the whole study population in the perspective of personalized approach of postural instability in ALS.

Statistical convergence for this model was achieved. The whole model effect was significant for postural instability discrimination *(P* < *0.0001) and useful (RSquare* = *0.6119)*, which supported the conclusion that the model significantly reflected the gait initiation variables globally by fitting the variables APA_amplitude_ and Braking-index. In addition, the test of lack-of-fit Chi-square for the whole model showed a large P value (*P* = *0.617*) and did not conclude that there was a significant lack of fit for this model. This supported the conclusion that the selected terms from the mixed stepwise selection provided enough information to the fitted model, and there was little to be gained by introducing additional terms in the model, such as using polynomials or crossed terms. This was corroborated with the significant contribution of each of the effects of APA_amplitude_ and Braking-index to the fit of the model (*P* = *0.0016 and 0.0005, respectively*). This shows that the biomechanical parameters APA_amplitude_ and Braking-index each contributed significantly and usefully to the improvement of the model’s performance for postural instability discrimination.

The regressors for APA_amplitude_ and Braking-index were negative, indicating that as the APA_amplitude_ or braking-index decreased, the probability of postural instability increased. Thus, for a 10 mm decrease in APA_amplitude_ or a 10% decrease in Braking-index, the odds of postural instability were 11.4 and 11.3 times, respectively, higher than the odds of no postural instability.

The area under ROC curve (AUC), referred to as an index of accuracy, is a measure of performance for a ROC curve. It was used to quantify how accurately the classifiers APA_amplitude_, Braking-index and PI_th_ can discriminate between the state of two patients, referred to as "postural instability" and "no postural instability". It was the probability that a subject with postural instability, randomly chosen, is ranked as more likely to have postural instability than a subject without postural instability randomly chosen, based on nonparametric Mann–Whitney U statistics used in calculating AUC.

The theoretical discrimination index of postural instability (PI_th_) showed the highest discriminatory performance (*AUC* = *0.953*). It significantly increased the sensitivity, that is the probability that the classifier correctly discriminates a postural instability condition that exists, while maintaining the specificity, which is the probability that the classifier correctly discriminates that a condition of postural instability does not exist. This was consistent with the aim of our study. Indeed, a false negative discrimination may have different consequences than false positive ones. Increasing sensitivity for postural instability discrimination has the advantage of initiating appropriate treatment and rehabilitation in a greater number of ALS patients who will have a condition of postural instability. This will also lower the possibility of falls and fractures with the acceptable risk of initiating treatment and rehabilitation in some patients who would not have needed it due to false positives. Moreover, maintaining specificity has the advantage of not increasing the unacceptable risk of not starting a rehabilitation program in some patients who will have a postural instability condition, due to false negatives. Not treating those patients who would have needed it would imply serious and costly consequences due to falls and fractures, impacting the quality of life.

Throughout our study, the theoretical discrimination index (PI_th_) reliability depended on the reliability of the pull test and gait initiation biomechanical data. Variability in pull test performance can lead to inadequate evaluation of postural instability^27^. Indeed, a study^27^ on postural instability assessment in Parkinson's disease reported that specific aspects of pull test were incorrectly performed regarding the position in 27.3%, strength and briskness of the pull in 84.9%, examiner's response in 36.4%, and technique issues in 9.1%. This study reported^27^ that 77.3% of patients with early Parkinson's disease were pulled too lightly, underestimating the deficit with possible bias in the score, especially between 0 and 1. Furthermore, the number of steps backward to differenciate a normal recovery response from retropulsion was not being clearly defined, postural stability score was not easily interpreted, especially to differentiate the scores 1 and 2^27^.

In our study, neurological assessment including pull test execution was standardized and performed by the same highly experienced neurologist examiner (PFP). For a successful execution of the pull test, the patients had sufficient space to move backward and assume an upright position that enabled a rapid corrective repositioning. Doing so, this avoided any attitude that could neutralize the pull before it was carried out, such as leaning forward or excessively widening the position of the feet. Consequently, the pulling force had not subjectively anticipated the postural instability degree. It was suitably adapted to the height and weight of the subjects to move the CoM to the point of compromising postural stability and requiring corrective trunk movements with a maximum of two steps backward for recovery^27^. Thus, the reliability between the pull test and K-means clustering of subjects across gait initiation biomechanical variables and postural instability was complete. This represented the extent to which the data collected in the study constituted correct and reliable representations of the variables measured.

During gait initiation, the anticipatory postural adjustments induce the CoP displacement backward relative to CoM, and toward the swing leg, which generates an imbalance which allows to initiate the CoM forward movement from the static posture^30,31^. The CoM forward movement is oriented in the direction of the stance leg^32^ to counterbalance prior to step execution^24,33,34^. Thus, the magnitude of imbalance plays an essential role in the regulation of global gait initiation kinematic^24,30,31^, as it creates the anticipatory conditions to propel the CoM forward^19,23^ while maintaining the stability during the step execution^35–37^. Our study showed that the CoP backward displacement, APA_amplitude_, was significantly associated with the motor performances in terms of step length and CoM forward progression velocity, and with the occurrence of postural instability in ALS patients. The shorter the magnitude of backward displacement of the CoP, the lower the step length and forward progression velocity, and the greater the risk of postural instability.

During step execution, the CoM is propelled forward to step forward, and out of the base of support^23,38^. The base of support is reduced by the act of lifting the swinging foot from the ground. The gap between the CoM and the CoP generates a disequilibrium which accelerates the CoM movement forward and downward^39^ in the direction of the swing leg. The CoM vertical downward velocity is actively braked before the foot contact with the ground^33,40^ by the increase in antigravity activity of the stance leg soleus muscle^30,31^. This reduces the bodily stress of the impact of the swing leg with the ground and maintains postural stability during the subsequent double stance phase^41^. Our study showed the CoM active vertical braking, or Braking-index, was significantly associated with the occurrence of postural instability in ALS patients. The greater the deterioration in active vertical braking, the greater the postural instability risk.

Thus, theoretical discrimination index (PI_th_) combines the CoP backward displacement (APA_amplitude_) and CoM active vertical braking (Braking-index), which were independent underlying biomechanical mechanisms of gait initiation, significantly involved in dynamic postural control and the occurrence of postural instability in ALS patients.

In our study, the gait initiation impairment in ALS patients did not stem from a decrease in muscle strength of the lower limbs, as the manual muscle tests revealed no significant differences between subjects with and without postural instability. However, patients with postural instability had increased muscle tone which can contribute to slow the CoM forward propulsion and deteriorate the dynamic postural stability during gait initiation^11^. Some studies showed in patients with progressive supranuclear palsy^41^, or Parkinson disease^42^ that increased muscle tone with stance leg muscle stiffness can impairegait initiation, and be partly responsible of an unstable state.

A limitation of this study was that we excluded patients with motor deficit in the lower limb that is a major cause of postural instability in ALS patients. We focused on other underlying mechanisms that are possibly of extrapyramidal^5,10^, cerebellar^14^, and vestibular^15^ origin. Consequently our model is applicable only in patients with a deficit limited in the upper limb and/or bulbar regions. Another limitation was that our model did not allow to discriminate between an extrapyramidal or pyramidal origin of the instability, and that we lack longitudinal data to validate whether our model can predict the occurrence and frequency of falls as well as the pull test.

## Conclusion

The theoretical postural instability discrimination index, designed as an integrator of the gait initiation biomechanical processes, significantly involved in dynamic postural control, was reliable and effective with a significant increased sensitivity in ALS patients. This, without reducing its specificity, which is key in view of the consequences of instability in terms of falls and fractures with repercussions on the quality of life. The importance of inter-method reliability between pull test and cluster analysis across the gait initiation and postural instability variables, represented the extent to which methods of data collecting in the study constituted correct representations of the variables measured.

This work is of significant clinical interest by offering an objective and measurable clinical index of physical and pathophysiological processes of postural instability in ALS. This may also be a useful complementary expertise in these patients for a personalized approach to postural instability.

## Abbreviations

ALS: Amyotrophic lateral sclerosis
APA: Anticipatory postural adjustment
PI_th_: Theoretical postural instability discrimination index
CoM: Center of mass
CoP: Center of pressure
